# Towards a model of resilience for transnational families of Filipina domestic workers

**DOI:** 10.1371/journal.pone.0183703

**Published:** 2017-08-24

**Authors:** Melissa R. Garabiles, Mira Alexis P. Ofreneo, Brian J. Hall

**Affiliations:** 1 Department of Psychology, Ateneo de Manila University, Quezon City, Philippines; 2 Global and Community Mental Health Research Group, Department of Psychology, Faculty of Social Sciences, University of Macau, Taipa, Macau (SAR), People’s Republic of China; 3 Department of Health, Behavior & Society, Johns Hopkins Bloomberg School of Public Health, Baltimore, Maryland, United States of America; Technion Israel Institute of Technology, ISRAEL

## Abstract

Many Filipinos experience poverty and poor employment opportunities. In order to alleviate poverty and provide sufficient resources for their families, numerous mothers leave the Philippines to become domestic workers. The present study aimed to build a model of family resilience for transnational families. A total of 33 participants consisting of Filipino transnational families, domestic workers, and key informants participated in a series of focus group discussions and interviews. A new model of resilience among transnational families of Filipina domestic helpers was created using a constructivist grounded theory approach. The model highlighted how temporal and spatial elements are embedded in collective migration experiences. Family narratives begin with the sacrifice of separation, where mothers leave their families for a chance to solve economic problems. To successfully adapt to their separation, the families undergo five relational processes. First, families communicate across space using technology to bridge relational distance. Second, families restructure across space through role sharing and the validation of each other’s efforts in their family roles. Third, families rebuild ties through temporary family reunification that bridge physical and relational distance. Fourth, families have the collective goal of permanent family reunification by ending migration to become complete again. Fifth, they strive to commit to their families by prioritizing them instead of succumbing to difficulties. Family resilience for transnational migrants is a collectivistic process, negotiated by each family member.

## Towards a model of resilience for transnational families of Filipina domestic workers

The Philippines is ranked among the top 10 countries with the largest population of migrants [1]. Since the 1970s, the Philippine government has institutionalized the deployment of Filipinos abroad, as it passed laws and regulations, created policies, built structures, and implemented programs and services for Overseas Filipino Workers (OFWs) and their families [2,3]. What started out as a temporary solution to problems in unemployment, underemployment, and foreign currency became a decades-long tradition [4]. Currently, there are 10.2 million OFWs working across the globe [5]. This means that in a population of almost 101 million [6], about 10% are working abroad. Their remittances account for more than 12% of the country’s GDP [7].

About half of all OFWs are women [8]. Their occupations vary but they are usually employed in service-oriented jobs. Some are professionals who work in hospitals and other health facilities as nurses and caregivers, some work in business settings as clerks and sales workers, and some work in their employers’ homes as domestic workers [4].

This study focuses on domestic workers, defined as persons who work in or for a household or households [9]. While this group does not represent all female OFWs, it does cover a significant portion of Filipino migrants. From 1992–2010, there were 1.2 million new domestic workers who left the Philippines, which comprised one-fourth of all new OFWs during that period [10]. Poverty and lack of job opportunities in the Philippines is associated with this trend [11]. Compared with other OFWs, domestic workers usually come from lower income groups in the Philippines [12]. Nonetheless, they are educated. In a survey of 211 departing domestic workers [13], the majority had high school diplomas, and about 20% had college degrees. However, in a country with a 6.6% unemployment rate and 16.3% underemployment rate [14], regular jobs that pay enough to sustain families’ needs may be hard to find. Thus, against this backdrop, these women’s migration is considered the best, if not the only chance for these families to escape economic adversities and seek upward social mobility [11].

Domestic workers are among the most marginalized and vulnerable groups in the world. Domestic work is low-skilled, undervalued, and largely unprotected from exploitation and human rights abuses [11,15]. It should be noted, however, that domestic workers are in a variety of contexts, which in turn affect their experiences. There are countries that have more legislation that promote their rights. Some high-income countries (i.e., Australia, Canada, USA) and countries in Latin America, the Caribbean, and Africa have legislation that provide domestic workers the same minimum protections accorded to other workers. Hence, domestic workers in these countries are more protected in terms of having limits to weekly normal hours of work, weekly days off from work and annual leaves, and comparable salaries with non-domestic workers. However, their counterparts in Middle Eastern and Asian countries may have fewer protections. Thus, these domestic workers are more susceptible to abuse in terms of excessive working hours and low wages [9].

Migration affects not just the domestic helpers but their families as well. Existing studies on Filipina domestic helpers and their transnational families have focused on the effects of separation and ways of coping with migration [i.e., 16–18]. Separation, continued poverty, and mothers’ post-departure stressors [19] create a context of multiple adversities that families must overcome. At present, little is known about factors that promote family resilience within the context of transnational migration and how families maintain their connection despite these multiple adversities. Family resilience is defined as interactional processes that enable families to overcome adversities and prolonged hardships [20]. This strengths-based approach posits that families can survive distress [21] and can cope and adapt successfully [20] even to chronic stress, to reclaim a healthy equilibrium [22].

This study aims to create a new model that encapsulates the process of family resilience among transnational families of Filipina domestic workers in the Macau Special Administrative Region, People’s Republic of China. Macau is home to 25,015 documented Filipino non-resident workers, more than 11,000 of whom are Filipina domestic workers [23]. This study seeks to contribute to the limited research literature on family resilience among transnational families. Most research has focused on individual rather than family and community resilience, which severely limits our conceptual and theoretical understanding of these processes [22,24].

### Multiple adversities for transnational families

Out-migration of a female primary caretaker is associated with stress and adversity experienced by her and her entire family [25]. First, migration destabilizes a family’s ability to maintain close relationships due to chronic physical distance [26]. To adjust, family members communicate with one another using technology [27–28], such as phone calls, Skype, social networking, and email messages, allowing the mothers to parent from afar and the fathers and children to send updates [17,27,29–32]. Consequently, a polymedia environment allows for continued expression of care and presence despite distance [33]. Further, family members reestablish and strengthen family connections during mothers’ visits [15,34–35]. Visits also allow mothers to see improvements at home and in their family’s overall economic status [35]. However, regular communication and visits are hindered by insufficient financial resources [15,17,34,36]. Compared to other occupations, domestic work is one of the lowest paid as domestic workers earn half or even as low as 20% of the average wages in the labor market [37]. Given low financial resources, some domestic workers may not be able to afford calls home and may not have access to reliable Internet either in their receiving country or with their family members back home. They may only see their family members once per year or every other year [38] and typically for no longer than 10 days. Prolonged physical distance makes restoring relationships difficult [15,39]. These families also have limited chances for reunification in the receiving countries because domestic workers are less likely than other workers to obtain permanent residency [36,40].

Graver disruptions in caregiving among mother-away families occur compared with father-away families [26] because of greater divergence from traditional Filipino gender roles of a maternal caregiver and a paternal provider [36,41]. When mothers leave, they shift their direct childcare responsibility from their own families to families in affluent countries, consequently creating a caretaking void for their own children [42–43]. This void, however, may be short-lived as some fathers actively parent their children [17–18,39–40,44–45]. In fact, studies have shown that 50% [26] to as high as 59.6% [46] of mother-away families report that fathers are the children’s main caregivers. While fathers may experience difficulty executing dual parenting roles [16–17,26], they normally receive support from their elder children. Children help with the household chores [29] and in taking care of their younger siblings [26,47]. The support they provide not only contributes to their families, but also provides them lessons in becoming responsible [26]. However, some children feel overburdened with the extra work [17].

In addition, Filipinos have a strong kinship system where extended family members permeate the daily lives of nuclear families through the provision of material and emotional resources [45,48,49]. When mothers migrate, relatives, particularly female relatives, provide childcare especially in tasks like teaching the children and nurturing them [16,26,46]. Children, in turn, are expected to allow their relatives to help care for them [47]. The provision of care from extended kin networks are likely to influence whether a family is resilient during transnational migration.

Second, family members experience psychological distress. This is expressed through feelings of loss, incompleteness, and sadness [16,38–39]. Mothers feel homesickness and worry about their left-behind families’ well-being and financial situation [15,34]. They also feel guilty for being physically away from their families [39].

Fathers may feel ashamed for not meeting societal standards of masculinity because they cannot provide enough for their families [16,18] and may experience role strain [18]. While some fathers still provide financially for their families [18,39–40], others may turn to vices and engage in extramarital affairs, which hinder them from rearing their children and maintaining positive ties with their wives [15–16,18]. In these circumstances, children yearn for more care [16,26]. They may feel abandoned and harbor resentment that their mothers left.

Third, domestic work does not provide sufficient economic resources. Domestic workers have low salaries and most of their earnings are remitted to the Philippines and used to buy gifts for their families [17,27]. They also have agency fees to pay and personal necessities to buy [19]. These make it difficult for families to pay off debts [34]. Some are in so much debt they end up as victims of debt bondage wherein they are coerced to work with little or no salary to repay loans [50]. At times, domestic workers become the sole providers because their husbands and children have become dependent on them for finances [38].

### Family resilience

There are existing models of family resilience. The systems theory of family resilience [24] conceptualizes family resilience as consisting of three key processes, namely, shared belief systems, organizational patterns, and communication processes. The family adjustment and adaptation response (FAAR) model [51] emphasizes the active process of balancing family demands or risk factors and family capabilities or protective factors to reach family resilience. Family demands include stressors brought about by changes, unresolved family strains, and daily hassles or minor life problems, whereas family capabilities include resources and family relationship patterns such as cohesiveness and flexibility.

These models, however, are not sufficient for evaluating resilience within transnational families given gaps in accounting for the spatial element in family resilience of transnational families and the temporal element in family resilience. The element of space differentiates transnational families from non-transnational families. The structure of transnational families is divided across physical space and national borders [39]. Taking into consideration the element of space means examining how these families overcome physical separation to remain intact.

The temporal element of family resilience highlights how processes unfold over time [20,24,52]. Families also need to adapt to a fluctuating array of adversities [24]. Some are normative transitions, like the initial phase of being apart and challenges in work and acculturative stressors. Some are unexpected and non-normative events, such as death or illness of loved ones and other traumatic events. In transnational families, the temporal element of family resilience manifests through the processes of redefining self, reconfiguring the family, and restoring relationships [39,53]. Thus, following the recommendations of Bonanno et al. [22], an exhaustive examination of the temporal element in family resilience would mean starting with a baseline of experiences when the families were still together in the Philippines, the adversities they experienced as transnational families, and followed by how they adapt to these adversities.

Aside from these gaps, existing literature on family resilience and migration only examines how individual members of the family respond to the problems they are experiencing [e.g., 54] and this might not be an appropriate or culturally informed approach. Individuals are nested within families and the relatedness between family members is a key determinant of well-being. This is especially true within collectivistic cultures. In the Philippines, familism or the value for family cohesion and being embedded in one’s family [55] is a predominant cultural value and is indeed related instrumentally to the sacrifice that women make to serve their families as OFWs. Further, examining multiple perspectives within families is needed to capture members’ unique contributions in overcoming adversities [21].

This study investigates family resilience in the context of migration, giving voice to key family members to account for multiple family perspectives and placing emphasis on spatial and temporal elements of their migration narrative. We created a model that contextualized the unique experiences of transnational families by incorporating the crucial elements of space and time. We used constructivist grounded theory approach. Grounded theory is designed to identify how a phenomenon develops, changes, or is maintained [56–58]. It is useful in generating a new theory which stays true to the data [59] and is specific to the given context [58]. As a constructivist approach, we co-constructed the resulting theory with the participants because we each played an active and integral role in shaping the research process and the findings [58,60].

## Method

Data collection occurred in two phases: a preliminary phase and main data collection phase. The preliminary phase aimed to obtain local definitions of resilience and to gather community nominations of resilient Filipina domestic workers’ families. This technique of using local definitions of constructs and community nominations in a grounded theory study was used in past research [i.e., 61]. The main data collection phase aimed to gather data about family resilience process from these families. All participants were Filipinos, based in either the Philippines or in Macau.

We obtained ethical approval from the university’s Research Ethics Committee. Before the interviews, all participants received written informed consents and assents. We assured them of confidentiality of information and that pseudonyms will be used in reports instead of their real names. We also instructed those whose family members participated in the study to inform us of any events that they did not want to be shared during their family’s group discussions.

A total of 33 people participated in our study. Of this number, 11 joined only the preliminary phase, 16 joined only the main data collection phase, and the remaining six participated in both preliminary and main data collection phases. All interviews and FGDs were conducted by the first author in Filipino and English.

### Preliminary data collection phase

The preliminary phase consisted of four focus group discussions (FGDs) and an individual interview. There was a total of 17 participants. Thirteen were domestic workers who participated in three of the four FGDs. The remaining four participants were Filipino key informants who had been providing services to Filipina domestic workers in Macau on a daily basis for an average of 3.25 years (*SD* = 2.20). Three of these key informants were priests who participated in the fourth FGD. One key informant was from a non-governmental organization who participated in an individual interview. Each FGD ran for one to two hours, with three to five participants per FGD, whereas the interview ran for one hour. FGDs and interviews were audio-recorded.

We asked participants about: (1) familial issues experienced by families of Filipina domestic workers in Macau; (2) description of families who resolved these issues the best; (3) description of families who resolved these issues the worst; and (4) specific families who fit the description of families that resolve issues the best. The second and third questions pertained to local definitions of resilience, specifically of families who were or were not resilient, whereas the fourth question aimed to get nominations for the main data collection.

The participants described resilient families as those who communicate and make plans together, families where husbands and wives strive to be faithful to one another, and families where fathers take care of their children. In contrast, non-resilient families do not solve their problems anymore. Instead, at least one of the members engage in vices (e.g., problematic gambling and alcohol misuse). The husbands and/or wives look for other partners. They forget about their marital commitments and do not make attempts to repair their relationship anymore, which leads to separation. The participants acknowledged, however, that those who are separated can still show resilience, albeit individual resilience.

Local definitions of resilience were used to identify families for in-depth interviews and FGDs. Participants nominated families of domestic workers who were resilient by providing the domestic worker’s name and contact information. They nominated six families: three nominees came from the key informants and three came from self- and peer-nominations among domestic workers. Five families agreed to participate in the main data collection phase. One nominee declined because of difficulties scheduling the interviews.

### Main data collection phase

The main data collection phase consisted of interviews (*n* = 22) and FGDs (*n* = 5) with: (a) family members from the five resilient families, and (b) family members from non-resilient families who were not nominated in the FGDs and did not fit the local definitions of family resilience. The mothers from non-resilient families were participants in the preliminary phase except for two mothers: one was recommended by a resilient mother and the other was a participant in another study. All interviews were audio-recorded.

Each resilient family consisted of a mother who was a domestic worker in Macau, a father based in the Philippines, and a child, for a total of 15 participants (Table 1). Each family had two to four children (*M* = 3.00, *SD* = 1.00). The children interviewed were chosen because they were willing and available for the interviews and lived with their fathers.

**Table 1:** Demographic information about resilient families.

	*N*	Age Range (*M*, *SD*)	Highest Educational Attainment
Mothers	5	34–44 (42.60, 4.83)	2 with 2-year college diploma, 3 with some college
Fathers	5	39–51 (45.00, 4.42)	1 with college degree, 2 with some college, 1 with vocational diploma, 1 with high school diploma
Children and adolescents	5 (4 females, 1 male)	11–20 (14.20, 3.56)	4 currently in grade school, 1 currently in college

We interviewed three members from the same family to ensure that the data represented multiple perspectives. The interviews with mothers were in person. The interviews with fathers and children were conducted through Skype calls. The interviews lasted between 0.75 and 2.75 hours (*M* = 1.43, *SD* = 0.72). Three of the five families also engaged in FGDs via Skype calls to provide supplemental data about collective family experiences of adversities and resilience, each FGD lasting between 1.25 and 1.50 hours (*M* = 1.33, *SD* = 0.14).

We gathered data from five non-resilient families to serve as natural comparators to evaluate whether the emerging concepts and categories were indeed important to our model [32,35]. This approach enabled us to utilize a comparative analytic approach, which provided greater clarity regarding the boundaries of resilience which we acknowledge is a process. We also wanted to increase the validity of our data to ensure that our results highlight resilience rather than normative family processes. Three of the non-resilient participants were from one family, consisting of a mother, father, and child. Each of them took part in individual interviews. There were also two FGDs between mother-child and mother-father. Four of the non-resilient participants were domestic workers who were interviewed individually. The interviews ran between 1.25 and 1.5 hours each (*M* = 1.38, *SD* = 0.14). The interviews with mothers were in person, whereas the interviews with fathers and children were through Skype (Table 2).

**Table 2:** Demographic information about non-resilient families.

Pseudonym	Age (in years)	Rationale for selection
Mrs. Diño	51	Infidelity of husband; limited and poor communication with 2 of 3 children
Mrs. Peña	54	Infidelity of husband
Mrs. Seña	48	Infidelity of husband
Mrs. Tiñio	37	Infidelity of husband and wife; limited communication with all children
Guaño Family		
Mrs. Guaño	41	Financial disputes; marital disputes on custody of son
Mr. Guaño	42	
Son (resided with his maternal grandmother)	8	

For the main data collection, the interview questions for both resilient and non-resilient families were asked in chronological order. The first question was about family life when the mother was still based in the Philippines, followed by the families’ immediate experiences after the mother left. They were then free to choose other family events to share. Afterwards, they were asked about their current family experiences. During the FGDs, we discussed key events that the family members shared during their individual interviews such as their life before the mother left to go abroad and their experiences during the mother’s visits. They talked about conflicts that occurred during those times and how they resolved these conflicts.

All data were transcribed by research assistants who were native Filipino and English speakers. Transcriptions were then checked for accuracy. Exemplars of themes included in this report were translated from Filipino to English.

### Data analysis and validity checks

We followed grounded theory method’s three-stage process: open coding, selective coding, and theoretical coding. In open coding, we took a bottom-up approach by identifying segments of text that could be coded into meaningful units for analysis. We grouped codes that comprised broader concepts, then we grouped these concepts into broader categories [60,62]. Using constant comparative analysis, we noted relations among the emerging categories, relations among the categories and their subcategories, the conditions for the emergence of the categories, and the consequences or outcomes per category [60]. We also conducted memoing by writing the tentative theory [63] and by keeping a record of personal thoughts throughout data analysis [64].

In selective coding, another round of constant comparison and memoing ensued to produce more saturated core categories. We also made diagrams to visualize the emerging relations [60]. In theoretical coding, we refined the core categories and cross-referenced them with existing literature to derive basic social processes that we turned into a theoretical model of family resilience [62].

This 3-stage process was nonlinear. We conducted over 15 iterations of data analysis and model comparison before arriving at the final model. To do this, we conducted cycles of induction and deduction to test concepts and their relationships with each other [65]. We checked if the interim theory confirmed or negated data from non-resilient families to check if they aligned or not aligned with the emerging theory. Particularly, we checked which codes, categories, and relationships among categories were present or absent in the narratives of resilient and non-resilient families. Saturation was reached after analysis of data from the five resilient families and from three non-resilient mothers.

Actual coding was conducted by the first author with consultations with the second and third authors throughout analysis. We also consulted four independent researchers to check their understanding of the emerging model. These led to improvements in category labelling, relating these categories with each other, and matching the interim diagram and model. The consultations also helped garner more objective views and diminish personal influence on the analysis. We also kept a paper trail of the data collection and analyses for documentation and as evidence of the link between the data and final report [66].

## Results

The narratives of resilient families start with experiences of economic adversities while they are together in the Philippines. To survive, mothers leave to work as domestic workers abroad.

To overcome the pain of separation, resilient families successfully engage in five relational processes. They engage in family communication across space to bridge the relational distance. They also undergo family restructuring and role validation across space. Family restructuring involves role sharing, wherein all the members make contributions based on their own capacities to fulfill essential family roles.

Resilient families rebuild ties through temporary family reunification, which occur during family visits when the mother returns home after long periods of separation. This process entails bridging both physical and relational distance to create moments when space is no longer a factor. These families also share the goal of permanent family reunification. They have a collective goal of ending their migration story so that they can be a complete family again in the future. Hence, for these families, migration is impermanent; the sacrifice of being separate is transient and finite.

**Fig 1:**
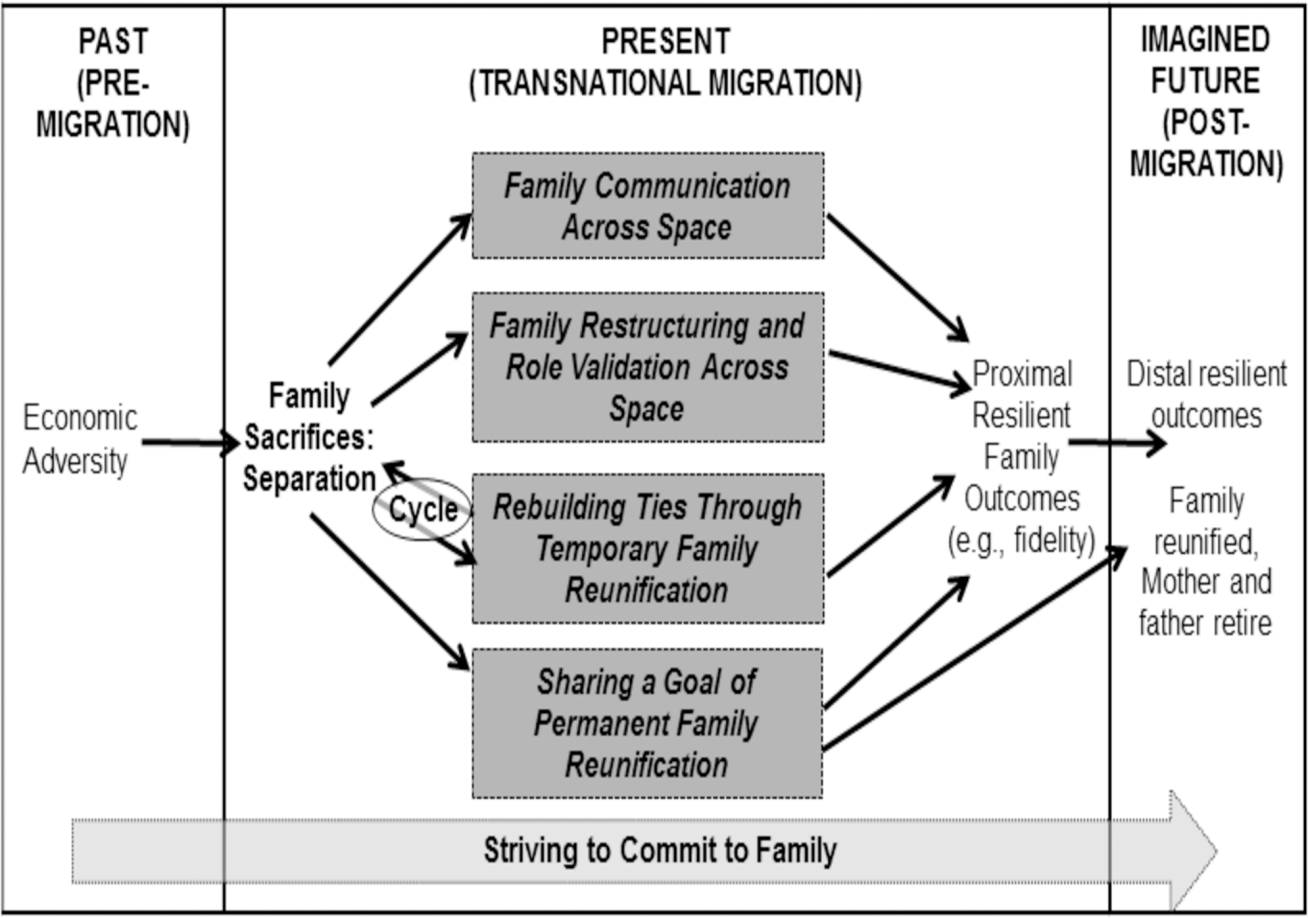
Model of resilience for transnational families of Filipina domestic workers.

Finally, resilient families strive to commit to family. The abovementioned relational processes are not easy because these happen amidst various difficulties. However, they strive to commit to their families by putting their family first through their sacrifice and collective problem solving (Fig 1).

### Family sacrifice of separation

All resilient families had extreme difficulties paying for their basic needs despite the mothers and fathers’ best efforts to work. Some children even helped out by selling food and crops. Entrenched in poverty, they have insufficient money to buy food and to pay for tuition fees and medical expenses. To survive, families resort to working multiple jobs, borrowing money, selling their livestock and farm land, and asking neighbors and relatives for food.

These economic adversities and the desire to do what is best for their family prompt families to break up physically by allowing the mothers to work abroad. Separation leads to mental distress, as families feel sad about being apart and worried about family members they cannot see. They also have strong feelings of longing to have a complete family again. Distress occurs over a long period of time and it begins from the moment the mother leaves and extends through the time they are apart, during brief visits, until reunification. The stories of the Mendez family, a resilient family, captured these moments:

Mr. Mendez: “It’s sad that my wife isn’t with us, right? My eldest son said, ‘Papa, if Mama is with us, we would be happier.’ ‘Yes, son,’ I said.” (Father, age 43).

Mrs. Mendez, on difficulty of going back to Macau after a vacation: “Here I go again (cries) … You were only able to be with your family for 10 days. Then you will leave them again. It’s really very hard, everyone really experiences that, I talk to other Filipinos, it’s really very hard.” (Mother, age 34)

Mr. and Mrs. Mendez’ eldest son: “I feel sad. … Because when she leaves, it’s right after New Year. After, after the fireworks, she leaves already.” (Child, age 12)

### Family communication across space

Migration inevitably creates physical distance between family members, which leads to loss of regular contact. To overcome this, resilient families engage in family communication across space through maintaining close ties and creating family memories. Families maintain close ties by talking to each other using technology to update each other about their daily lives and about important news. They also communicate to express positive emotions like love and care to each other and to help manage difficult emotions, such as sadness over family’s separation and listening and responding to each other’s grievances.

Resilient families also create family memories by celebrating special events together over video calls, cellphone calls, or social media updates. Hence, technology is able to bridge families together during special moments.

Family communication across space quells feelings of sadness and incompleteness. Instead, resilient families experience relational benefits of feeling loved and cared for despite their physical distance. They continue to be a unified family because they keep current with each other’s lives. Hence, even if they are apart physically, they are still connected relationally. Mr. Torres, a member of a resilient family, illustrated this during his interview:

“We’re apart but it’s like we’re still near each other. Like we didn’t separate because we get to talk.” (Father, age 51)

For family communication across space to occur, all the members of the family need to exert effort to sustain open communication. Lack of open communication exacerbates the emotional pain of physical distance because it adds relational distance. The lack of effort is shown in the story of Mrs. Diño, one of the mothers in a non-resilient family. While in Macau, Mrs. Diño exerted effort to contact her children but they did not share pertinent details about their lives, including the news that one of her sons was in an accident:

“He said, ‘You take care of other children. Then, when we get sick, no one takes care of us. Did you know that I met an accident? Where were you?’ It’s so painful (cries) … [I said,] ‘Why didn’t you tell me? I call you, I check on you, why didn’t you inform me? Do you know what you’re doing to me? You’re taking away my right as a parent!’ (cries). (Mother, age 51)

### Family restructuring and role validation across space

When mothers leave to work abroad, the customary familial arrangement of a male breadwinner and female caretaker is disrupted, leading to problems in fulfilling familial roles. To address this, resilient families engage in family restructuring and role validation across space. In family restructuring, the family members share in the fulfillment of the familial roles of a provider, caretaker, and marital partner. In terms of the provider role, the mother becomes the new main provider because she sends remittances and packages to her family regularly, hence lessening debts and allowing the family to meet its daily needs more easily. The father also contributes financially by working to augment his wife’s income and optimizing the remittances by budgeting them well. The children contribute by being responsible with their allowance. Children who are old enough also work to earn money to spend for school expenses and even household expenses.

In terms of the caretaker role, it is the father who takes on the role of primary household and child caretaker. The father is hands-on with the children, especially when they were still young. Aside from the father, the children contribute by taking care of themselves more, taking on some household chores, and helping to take care of each other. Moreover, the mother is still able to execute her mothering role by communicating with her children using technology.

The families recognize receiving help from their relatives, especially the in-laws. Relatives provide financial support during times of emergency or when the mother is unable to send remittances on time. Relatives also provide childcare support when the father leaves the home to work or to run errands. While their support is important, the mothers and fathers report that they prefer that the father takes over the reins, as he is capable to do the mother’s tasks in housekeeping and childrearing. Further, this setup limits the influence of relatives who may overstep their boundaries by spoiling the children or gossiping about the mother or the father.

In terms of executing the marital role, the mother and the father remind each other to stay faithful to one another and to focus on having a complete family to alleviate doubts about infidelity. The couple also avoids listening to gossip about infidelity because they know that gossip disrupts their faith in each other.

Family role validation also takes place, which involves appreciating each other’s contributions in fulfilling family roles and acknowledging that their efforts have been indispensable to their family. An FGD with the Reyes family, a resilient family, serves as an exemplar:

Mr. Reyes to his wife: “I am thankful to her because what I am unable to give my family, she is able to find a way to give. … Like financially, because she’s the one there right now to be the breadwinner of the family.” (Father, age 46)

Mrs. Reyes to her husband: “I am thankful that you did not look for another woman! (laughs) … Because most here, most of the OFWs here, in less than a year, their families are broken already. But that’s why I’m so proud of you because you stayed strong, you stayed strong, you didn’t neglect your obligation as the head of the family … You are facing your responsibilities to our child, that you support her, you correct her ways, you’re there to assist her. That’s why I salute you that you didn’t neglect our daughter ever since she was small.” (Mother, age 45)

Mrs. Reyes to her daughter: “Continue all the good things you are doing and may the Lord have mercy on you so that you can finish [studying] already.” (Mother, age 45)

Family restructuring and role validation lead to resilient outcomes. Sharing responsibilities allows the family members to feel relief and enhances their ability to execute their roles. There are financial benefits because they are better able to meet daily needs, to go to school more regularly, and to start making investments. There are also relational benefits of feeling each other’s presence, love, care, and value in the family. Members of resilient families expressed these outcomes:

Mr. Gomez: “I’m happy when my child is happy. Because it sometimes helps, her package helps. To avoid feeling homesick.” (Father, age 39)

Mrs. Gomez: “My family tells me that my husband really loves my child. So I don’t have worries, I don’t have worries that my child is being neglected. None.” (Mother, age 44)

Mr. and Mrs. Cortes’ daughter, regarding food sent by her mother: “It makes me feel lighter, it’s like she’s feeding, she’s feeding us already.” (Child, age 11)

For family restructuring and role validation to take place, the family members should share the role that is traditionally theirs and allow other members to make contributions. Family members should also accept and fulfill these roles. If a member does not share or fulfill a role, restructuring does not happen. This is what transpired with Mrs. Tiñio, a mother in a non- resilient family who blamed herself for being an inconsistent provider when she became involved with another man while she was abroad:

“I sent remittances but only rarely … I focused on having a relationship, I focused on my feelings to him. Like I forgot that I had a family.” (Mother, age 37)

The lack of the process of family restructuring and role validation across space is also evident in the stories of the Guaño family, a non-resilient family. Mrs. Guaño gave custody of her son to her mother because she believed the gossip that her husband was neglectful. However, Mr. Guaño contended that he is an involved father:

“I told my wife, ‘Why? I’m here, why would I neglect my child? He’s my child,’ I told her. Then my mother-in-law told me, ‘Can you feed him?’ Her mother asked me. ‘I can, he’s my child, I would not neglect him,’ I told her.” (Father, age 42)

### Rebuilding ties through temporary family reunification

Physical separation threatens close relations between the members of transnational families. These families are rendered incomplete and broken with the mothers’ absence. To adapt, resilient families rebuild ties through temporary family reunification. It is a bittersweet process that enables them to make up for lost time and be a complete family amidst the impending pain of separating again because the mothers have to go back abroad to work. This process involves getting to know each other again by sharing stories with each other on how their lives have been. They resume old roles and practices, such as going out and celebrating events together. The mother is able to take care of her children directly, express her affection, and do special things for them. This was shared by Mr. and Mrs. Torres’ daughter, who recalled how her mother surprised them with a visit after being apart for five years:

“When I got home, there was someone sleeping on the bed. … When I looked closely, it was my mother. Son of a fish. Nothing, I just felt shocked. I said, ‘You’re so annoying!’ Then I hit her repeatedly, I was crying, I don’t know why. There, I’m going to cry again (cries).” (Child, age 20)

The father and the children are able to express their affection by giving the mother special treatment. They hug her, kiss her, and cook for her. Furthermore, the mother and father are able to rekindle their relationship by being sweet to each other, having sex, expressing each other’s importance, and discussing and resolving issues like finances, doubts about infidelity, and for some, actual infidelity.

Rebuilding ties provides the relational benefit of experiencing being complete again, which strengthens the relationship even when the family separates again. The chance to update each other also allows for greater understanding and appreciation of each other’s efforts and sacrifices. Furthermore, the family becomes hopeful that reunification will happen again in the future.

For the couple, rebuilding ties removes doubts about infidelity and instead increases trust. For instance, Mrs. Torres reconnected with her husband sexually during her visit, which consequently improved their marital relationship:

“Maybe he learned that I am good at having sex (laughs). Maybe after 5 years, he did not doubt anymore. Because they say it’s through sex that the two of you, that the man will know if someone else had sex with his wife or not. Ah! They will feel if they were cheated on or not.” (Mother, age 45)

For rebuilding ties to occur, all the family members need to exert effort to be complete momentarily. The mother needs to try to visit her family and the father and the children need to forego their usual routine to accommodate the mother’s return. This effort was shown by Mrs. Torres’ daughter, who, despite being a working student, tried to optimize her mother’s visit:

“She would keep on talking about all sorts of things. I was so sleepy already but I still talked to her because of course, I missed hearing her talk.” (Child, age 20)

### A shared goal of permanent family reunification

Resilient families share the goal of permanent family reunification. Achieving this goal is predicated on first improving family finances. To do this, the mother and the father commit to being excellent employees. They also start to invest or to plan to invest their money.

For resilient families, migration is merely a temporary condition they will eventually overcome, starting with the children’s graduation from college which marks the beginning of the end of the mother’s migration. The family dreams that the mother will come back for good and will never part from them again. Both parents will stop working and enjoy their retirement. The children will work for them as a way of giving back, expressing their love, and improving their lives. The son of Mr. and Mrs. Mendez shared the following:

“I will repay them for their past sacrifices for us. … I will make their lives comfortable. … I will show my love for them, I will work.” (Child, age 12)

As consequence, families feel a sense of hope for a better future. The adversities they face are impermanent and can be overcome. There is a sense of achievement as they move closer to realizing their dreams. These were exemplified by Mrs. Gomez, who shared how she feels when she leaves her family after a vacation:

“I’m also sad to leave, but that feeling of hope is still there. Because I know I’m going back abroad, when I get back, we will fulfill some of our plans. Something will be fulfilled. That’s what’s always, that’s always my purpose for coming back [to Macau], it is to take care of what I see is lacking in the Philippines. To take advantage of the chance that I am here. I go back so that come the time I want to go home, it will be for good.” (Mother, age 44)

A necessary condition is future orientation. For them, the period of being a transnational family is only the process toward the end goal of being a family. They need to see the potential for improvement and they need to want to be a reunited family in the future.

### Striving to commit to family

Family resilience processes are difficult to do. Family communication across space is met with high communication costs and lack of accessibility of technology. Family restructuring and role validation are challenged by difficulties enacting new roles. Rebuilding ties through temporary family reunification reignites feelings of loneliness with the looming separation. The goal of permanent family reunification is impeded by lack of money.

Nonetheless, resilient families strive to commit to their families throughout the migration process. They constantly weave together a shared collective narrative, starting with the acknowledgment that separation is a necessary sacrifice for the family. This acknowledgment is developed through discussions: the mother and father remind each other that migration is the best solution to economic difficulties; the parents talk to the children to help them understand that the mother is abroad for their sake.

Through these discussions, the children learn to acknowledge that separation is needed for their families to survive. An exemplar is Mr. and Mrs. Torres’ daughter:

“Other children rebel against their parents. Other children, if their parents are far, they get angry at them. They don’t know that their parents do that for their own sake. It’s how they think, that they get angry because the parents don’t, like there’s lack of love from them, like that, it’s all about the money. But it’s not like that. What they are doing is for you. Because of love, you understand?” (Child, age 20)

The children acknowledge that they themselves should act upon the pain of separation to lessen its effects. They make the commitment to stay a family by making their own sacrifices. For example, Mrs. Reyes shared that her children find ways to have money to contact her during her birthday:

“That’s what my husband said, ‘There,’ he said, ‘Your children sell vegetables just to have money to buy [cellphone] load so they can call you and tell you Happy Birthday.’ (laughs). There. ‘I love you Mama, happy birthday!’” (Mother, age 45)

Striving to commit to the family also means solving problems together. This is evident with how the Cortes couple dealt with issues of infidelity, wherein Mrs. Cortes ended her affair and then went home to fix her relationship with her husband. Mr. Cortes waited to talk to his wife instead of listening to gossips from relatives:

Mrs. Cortes: “‘Don’t mind it!,’ he said. ‘Just think that it was just a storm that passed us because you proved anyway that we still weigh more heavily in your heart.’ … His love, it was still there. That’s why (cries) My God! His support was really still there.” (Mother, age 45)

The families’ effort to commit to the family leads to relational benefits, such as showing understanding for one another and giving more effort into the relationship. This was experienced by the daughter of Mr. and Mrs. Reyes:

“Before, I didn’t feel good about my Mama. But now, I understand that I don’t see Mama because she wants to help us. We understand now that it’s so hard to be away from one’s children.” (Child, age 13)

To strive to commit to the family, each member needs to exert effort to solve problems together. This was absent in the experience of Mrs. Seña from a non-resilient family. She wanted to repair her troubled marriage with her husband who had an affair. Instead, her husband refused to talk to her, continued with his affairs, and started to gossip that she was having an affair:

“A woman sent me a message in Facebook. She told me, ‘My name is like this. I just want to confirm if you and your husband are really separated already,’ she said that. ‘That you already have a new husband who’s a foreigner based in America.’ They thought I was in America. Even his other woman thought I was in America. Then, I was just feeling ok. Then she said, ‘I’m pregnant. He is the father.’” (Mother, age 48)

When a family member does not commit to the family, physical distance is aggravated by relational distance. Thus, it is necessary for all the members to continually strive to put the family first to survive adversities brought about by migration.

## Discussion

Our current research shows the importance of family communication, family flexibility to rearrange structures and roles, and family cohesiveness or connectedness, which is consistent with previous models of family resilience, the systems theory of family resilience [20] and the family adjustment and adaptation response model [51]. However, the current study makes a unique contribution to what is known about family resilience for migrants by showing how these processes occur amidst the adversity of physical separation for long periods of time and the compounding effects of grinding poverty. Transnational families of Filipina domestic workers communicate, restructure, and commit relationally across borders and across time, while being continually stressed by difficulties in meeting daily needs.

The process by which a transnational family restructures is worth highlighting. Instead of restructuring through role reversal among mother-away families [18], resilient families in this study share breadwinning and caretaking roles, which helps relieve pressure and fosters a greater sense of family involvement. A critical finding therefore is that resilient families share responsibilities flexibly and realize that family roles can be fulfilled by many members. Sharing roles also distributes the responsibility among the members instead of attributing one role to just one member. This is in contrast to traditional precepts on assigning roles almost exclusively to just one family member and based on gender. Sharing roles prevents role strain which happens when members need to enact a role that is not traditionally theirs.

Resilient families recognized that extended families have helped them, and yet they still considered the fathers are the main caregivers. Separately, these findings relate well with existing literature on extended family members’ contributions within transnational families and collectivist cultures [16,26,29,45–46,48–49] and on left-behind fathers’ taking on caregiving tasks [i.e., 17–18,26,39–40,44–46]. Transnational families may assert the role of the father as primary caretaker as a preemptive measure to minimize problems with extended family members. Studies have framed these findings as the fathers’ way of reclaiming power after losing their breadwinning status, as they redefine their identity from breadwinners to men who may not be breadwinners anymore but are still responsible, productive, capable, and in control as manifested in their caregiving [18,67]. Moreover, these findings show the importance of investigating not just how extended family members may provide support to transnational families but also how they may bring about problems [i.e., 15]. As this current study has shown, extended families may incite conflict when they diverge from parents’ preferred ways of rearing children and when they gossip and spread false information about the family members.

The findings on rebuilding ties through temporary family reunification and sharing the goal of permanent family reunification processes contribute to the scant literature on reunification in migration studies [68]. The findings align with existing literature on the importance of the mothers’ visits in revitalizing family connections [15,34–35]. Rebuilding ties illustrates the dynamic situation of transnational families who are entrenched in a cycle of separation and temporary reunification. Resilient families have the collective and explicit goal of ending this cycle to emerge as a complete family in the end, whereas non-resilient families no longer express this desire. Sharing the goal of permanent family reunification makes migration bearable.

Moreover, striving to commit to family is reminiscent of familism. This study, however, emphasizes that remaining intact despite adversities is an ongoing process. There is the active and recurrent need to make the choice and the constant effort for each family member to remain connected even while apart.

### Limitations

This study was the first to investigate processes of family resilience among transnational migrant families. We analyzed the data using multiple informants with an emphasis on incorporating key temporal and spatial dimensions of resilience important to this community. We also utilized local definitions of resilience to inform our analysis. Through mapping family storylines following Bonanno et al.’s [22] recommendations, this study showed that family resilience among transnational migrants is a process that traverses time and space. That is, the model developed from qualitative inquiry illustrates families’ experiences of being together during pre-migration times, being apart at present, and being together in a post-migration future.

Despite these strengths, the current study had several limitations. First, data collected from the fathers and children were conducted via online method because they were located in different parts of the Philippines. This method caused some interruptions due to unstable Internet connection and may have limited the researchers’ ability to capture complete information from these informants. A more stable Internet connection would help in having clearer and smoother data collection. Second, our current level of analysis is focused on family resilience. Therefore, we were not able to investigate the extent to which the receiving country environment shapes these resilience processes. We also did not look into parenting styles which could describe how parents can best rear their children to promote resilience for the family. Third, we did not investigate communication styles, such as how the mothers are able to maintain their relationships from afar while navigating traditional Filipino gender roles.

Finally, since resilience is an ongoing process, it is possible that the families categorized as resilient may transition to non-resilient status if increasing stressors and demands are placed on the family. We were not able to identify whether such a tipping point existed within the non-resilient families or to capture the differences between these families regarding the number of stressors, time since migration, and relative poverty, which may help to account for resilience observed in our study. External factors and other salient family characteristics that may contribute to family resilience should be interrogated in future research.

### Conclusions and future directions

The current research informs several key research priorities for Filipino transnational families. First, the model proposes relationships between family resilience processes and resilient outcomes (e.g., reunification) which can be tested quantitatively to make predictions and generalizations about the population of transnational families of domestic helpers. Second, a longitudinal study would capture causal relationships and changes in adversities experienced and in how families adapt to these adversities. Third, the use of information and communication technologies is a key factor in facilitating transnational family resilience. Future research is needed to explore specific patterns of communication and usage that maximize transnational resilience.

Finally, the current findings have implications for intervention development. Following the timeline of the model which starts before the mothers’ migration, seminars before they leave can be conducted to help families create a collective narrative. The narrative includes how they will communicate from afar, how they will share familial roles, and plans to renew ties during visits and to reunite permanently in the future. Philippine government agencies can also create programs to educate families about financial literacy to teach them how to budget and save for the future. Fathers can be taught housekeeping and childrearing skills. Schools or communities can create support groups to assist fathers and children who are growing up without their partners and mothers. Through these interventions, the family could develop the competencies to execute resilient processes to facilitate successful adaptation.
